# The benefits of tourism for rural community development

**DOI:** 10.1057/s41599-023-01610-4

**Published:** 2023-03-31

**Authors:** Yung-Lun Liu, Jui-Te Chiang, Pen-Fa Ko

**Affiliations:** 1 Chienkuo Technology University, Changhua, Taiwan; 2 Dayeh University, Changhua, Taiwan

**Keywords:** Development studies, Business and management, Economics

## Abstract

While the main benefits of rural tourism have been studied extensively, most of these studies have focused on the development of sustainable rural tourism. The role of tourism contributions to rural community development remains unexplored. Little is known about what tourism contribution dimensions are available for policy-makers and how these dimensions affect rural tourism contributions. Without a clear picture and indication of what benefits rural tourism can provide for rural communities, policy-makers might not invest limited resources in such projects. The objectives of this study are threefold. First, we outline a rural tourism contribution model that policy-makers can use to support tourism-based rural community development. Second, we address several methodological limitations that undermine current sustainability model development and recommend feasible methodological solutions. Third, we propose a six-step theoretical procedure as a guideline for constructing a valid contribution model. We find four primary attributes of rural tourism contributions to rural community development; economic, sociocultural, environmental, and leisure and educational, and 32 subattributes. Ultimately, we confirm that economic benefits are the most significant contribution. Our findings have several practical and methodological implications and could be used as policy-making guidelines for rural community development.

## Introduction

In many countries, rural areas are less developed than urban areas. They are often perceived as having many problems, such as low productivity, low education, and low income. Other issues include population shifts from rural to urban areas, low economic growth, declining employment opportunities, the loss of farms, impacts on historical and cultural heritage, sharp demographic changes, and low quality of life. These issues indicate that maintaining agricultural activities without change might create deeper social problems in rural regions. Li et al. (2019) analyzed why some rural areas decline while others do not. They emphasized that it is necessary to improve rural communities’ resilience by developing new tourism activities in response to potential urban demands. In addition, to overcome the inevitability of rural decline, Markey et al. (2008) pointed out that reversing rural recession requires investment orientation and policy support reform, for example, regarding tourism. Therefore, adopting rural tourism as an alternative development approach has become a preferred strategy in efforts to balance economic, social, cultural, and environmental regeneration.

Why should rural regions devote themselves to tourism-based development? What benefits can rural tourism bring to a rural community, particularly during and after the COVID pandemic? Without a clear picture and answers to these questions, policy-makers might not invest limited resources in such projects. Understanding the contributions of rural tourism to rural community development is critical for helping government and community planners realize whether rural tourism development is beneficial. Policy-makers are aware that reducing rural vulnerability and enhancing rural resilience is a necessary but challenging task; therefore, it is important to consider the equilibrium between rural development and potential negative impacts. For example, economic growth may improve the quality of life and enhance the well-being index. However, it may worsen income inequality, increase the demand for green landscapes, and intensify environmental pollution, and these changes may impede natural preservation in rural regions and make local residents’ lives more stressful. This might lead policy-makers to question whether they should support tourism-based rural development. Thus, the provision of specific information on the contributions of rural tourism is crucial for policy-makers.

Recently, most research has focused on rural sustainable tourism development (Asmelash and Kumar, 2019; Polukhina et al., 2021), and few studies have considered the contributions of rural tourism. Sustainability refers to the ability of a destination to maintain production over time in the face of long-term constraints and pressures (Altieri et al., 2018). In this study, we focus on rural tourism contributions, meaning what rural tourism contributes or does to help produce something or make it better or more successful. More specifically, we focus on rural tourism’s contributions, not its sustainability, as these goals and directions differ. Today, rural tourism has responded to the new demand trends of short-term tourists, directly providing visitors with unique services and opportunities to contact other business channels. The impact on the countryside is multifaceted, but many potential factors have not been explored (Arroyo et al., 2013; Tew and Barbieri, 2012). For example, the demand for remote nature-based destinations has increased due to the fear of COVID-19 infection, the perceived risk of crowding, and a desire for low tourist density. Juschten and Hössinger (2020) showed that the impact of COVID-19 led to a surge in demand for natural parks, forests, and rural areas. Vaishar and Šťastná (2022) demonstrated that the countryside is gaining more domestic tourists due to natural, gastronomic, and local attractions. Thus, they contended that the COVID-19 pandemic created rural tourism opportunities.

Following this change in tourism demand, rural regions are no longer associated merely with agricultural commodity production. Instead, they are seen as fruitful locations for stimulating new socioeconomic activities and mitigating public mental health issues (Kabadayi et al., 2020). Despite such new opportunities in rural areas, there is still a lack of research that provides policy-makers with information about tourism development in rural communities (Petrovi’c et al., 2018; Vaishar and Šťastná, 2022). Although there are many novel benefits that tourism can bring to rural communities, these have not been considered in the rural community development literature. For example, Ram et al. (2022) showed that the presence of people with mental health issues, such as nonclinical depression, is negatively correlated with domestic tourism, such as rural tourism. Yang et al. (2021) found that the contribution of rural tourism to employment is significant; they indicated that the proportion of nonagricultural jobs had increased by 99.57%, and tourism in rural communities had become the leading industry at their research site in China, with a value ten times higher than that of agricultural output. Therefore, rural tourism is vital in counteracting public mental health issues and can potentially advance regional resilience, identity, and well-being (López-Sanz et al., 2021).

Since the government plays a critical role in rural tourism development, providing valuable insights, perspectives, and recommendations to policy-makers to foster sustainable policies and practices in rural destinations is essential (Liu et al., 2020). Despite the variables developed over time to address particular aspects of rural tourism development, there is still a lack of specific variables and an overall measurement framework for understanding the contributions of rural tourism. Therefore, more evidence is needed to understand how rural tourism influences rural communities from various structural perspectives and to prompt policy-makers to accept rural tourism as an effective development policy or strategy for rural community development. In this paper, we aim to fill this gap.

The remainder of this paper is organized as follows: the section “Literature review” presents the literature review. Our methodology is described in the section “Methodology”, and our results are presented in the section “Results”. Our discussion in the section “Discussion/implications” places our findings in perspective by describing their theoretical and practical implications, and we provide concluding remarks in the section “Conclusion”.

## Literature review

### The role of rural tourism

The UNWTO (2021) defined rural tourism as a type of tourism in which a visitor’s experience is related to a wide range of products generally linked to nature-based activity, agriculture, rural lifestyle/culture, angling, and sightseeing. Rural tourism has been used as a valid developmental strategy in rural areas in many developed and developing countries. This developmental strategy aims to enable a rural community to grow while preserving its traditional culture (Kaptan et al., 2020). In rural areas, ongoing encounters and interactions between humans and nature occur, as well as mutual transformations. These phenomena take place across a wide range of practices that are spatially and temporally bound, including agriculture, forestry, fishing, hunting, farm tourism, cultural heritage preservation, and country life (Hegarty and Przezbórska, 2005). To date, rural tourism in many places has become an important new element of the regional rural economy; it is increasing in importance as both a strategic sector and a way to boost the development of rural regions (Polukhina et al., 2021). Urban visitors’ demand for short-term leisure activities has increased because of the COVID-19 pandemic (Slater, 2020). Furthermore, as tourists shifted their preferences from exotic to local rural tourism amid COVID-19, Marques et al. (2022) suggested that this trend is a new opportunity that should be seized, as rural development no longer relies on agriculture alone. Instead, other practices, such as rural tourism, have become opportunities for rural areas. Ironically, urbanization has both caused severe problems in rural areas and stimulated rural tourism development as an alternative means of economic revitalization (Lewis and Delisle, 2004). Rural tourism provides many unique events and activities that people who live in urban areas are interested in, such as agricultural festivals, crafts, historical buildings, natural preservation, nostalgia, cuisine, and opportunities for family togetherness and relaxation (Christou, 2020; Getz, 2008). As rural tourism provides visitors from urban areas with various kinds of psychological, educational, social, esthetic, and physical satisfaction, it has brought unprecedented numbers of tourists to rural communities, stimulated economic growth, improved the viability of these communities, and enhanced their living standards (Nicholson and Pearce, 2001). For example, rural tourism practitioners have obtained significant economic effects, including more income, more direct sales, better profit margins, and more opportunities to sell agricultural products or craft items (Everett and Slocum, 2013). Local residents can participate in the development of rural tourism, and it does not necessarily depend on external resources. Hence, it provides entrepreneurial opportunities (Lee et al., 2006). From an environmental perspective, rural tourism is rooted in a contemporary theoretical shift from cherishing local agricultural resources to restoring the balance between people and ecosystems. Thus, rural land is preserved, natural landscapes are maintained, and green consumerism drives farmers to focus on organic products, green chemistry, and value-added products, such as land ethics (Higham and Ritchie, 2001). Therefore, the potential contributions of rural tourism are significant and profound (Marques, 2006; Phillip et al., 2010). Understanding its contributions to rural community development could encourage greater policy-maker investment and resident support (Yang et al., 2010).

### Contributions of rural tourism to rural community development

Maintaining active local communities while preventing the depopulation and degradation of rural areas requires a holistic approach and processes that support sustainability. What can rural tourism contribute to rural development? In the literature, rural tourism has been shown to bring benefits such as stimulating economic growth (Oh, 2005), strengthening rural and regional economies (Lankford, 1994), alleviating poverty (Zhao et al., 2007), and improving living standards in local communities (Uysal et al., 2016). In addition to these economic contributions, what other elements have not been identified and discussed (Su et al., 2020)? To answer these questions, additional evidence is a prerequisite. Thus, this study examines the following four aspects. (1) The economic perspective: The clustering of activities offered by rural tourism stimulates cooperation and partnerships between local communities and serves as a vehicle for creating various economic benefits. For example, rural tourism improves employment opportunities and stability, local residents’ income, investment, entrepreneurial opportunities, agricultural production value-added, capital formation, economic resilience, business viability, and local tax revenue (Atun et al., 2019; Cheng and Zhang, 2020; Choi and Sirakaya, 2006; Chong and Balasingam, 2019; Cunha et al., 2020). (2) The sociocultural perspective: Rural tourism no longer refers solely to the benefits of agricultural production; through economic improvement, it represents a greater diversity of activities. It is important to take advantage of the novel social and cultural alternatives offered by rural tourism, which contribute to the countryside. For example, rural tourism can be a vehicle for introducing farmers to potential new markets through more interactions with consumers and other value chain members. Under such circumstances, the sociocultural benefits of rural tourism are multifaceted. These include improved rural area depopulation prevention (López-Sanz et al., 2021), cultural and heritage preservation, and enhanced social stability compared to farms that do not engage in the tourism business (Ma et al., 2021; Yang et al., 2021). Additional benefits are improved quality of life; revitalization of local crafts, customs, and cultures; restoration of historical buildings and community identities; and increased opportunities for social contact and exchange, which enhance community visibility, pride, and cultural integrity (Kelliher et al., 2018; López-Sanz et al., 2021; Ryu et al., 2020; Silva and Leal, 2015). (3) The environmental perspective: Many farms in rural areas have been rendered noncompetitive due to a shortage of labor, poor managerial skills, and a lack of financial support (Coria and Calfucura, 2012). Although there can be immense pressure to maintain a farm in a family and to continue using land for agriculture, these problems could cause families to sell or abandon their farms or lands (Tew and Barbieri, 2012). In addition, unless new income pours into rural areas, farm owners cannot preserve their land and its natural aspects; thus, they tend to allow their land to become derelict or sell it. In the improved economic conditions after farms diversify into rural tourism, rural communities have more money to provide environmental care for their natural scenic areas, pastoral resources, forests, wetlands, biodiversity, pesticide mitigation, and unique landscapes (Theodori, 2001; Vail and Hultkrantz, 2000). Ultimately, the entire image of a rural community is affected; the community is imbued with vitality, and farms that participate in rural tourism instill more togetherness among families and rural communities. In this study, the environmental benefits induced by rural tourism led to improved natural environmental conservation, biodiversity, environmental awareness, infrastructure, green chemistry, unspoiled land, and family land (Di and Laura, 2021; Lane, 1994; Ryu et al., 2020; Yang et al., 2021). (4) The leisure and educational perspective: Rural tourism is a diverse strategy associated with an ongoing flow of development models that commercialize a wide range of farming practices for residents and visitors. Rural territories often present a rich set of unique resources that, if well managed, allow multiple appealing, authentic, and memorable tourist experiences. Tourists frequently comment that the rural tourism experience positively contrasts with the stress and other negatively perceived conditions of daily urban life. This is reflected in opposing, compelling images of home and a visited rural destination (Kastenholz et al., 2012). In other words, tourists’ positive experiences result from the attractions and activities of rural tourism destinations that may be deemed sensorially, symbolically, or socially opposed to urban life (Kastenholz et al. 2018). These experiences are associated with the “search for authenticity” in the context of the tension between the nostalgic images of an idealized past and the demands of stressful modern times. Although visitors search for the psychological fulfillment of hedonic, self-actualization, challenge, accomplishment, exploration, and discovery goals, some authors have uncovered the effects of rural tourism in a different context. For example, Otto and Ritchie (1996) revealed that the quality of a rural tourism service provides a tourist experience in four dimensions—hedonic, peace of mind, involvement, and recognition. Quadri-Felitti and Fiore (2013) identified the relevant impact of education, particularly esthetics, versus memory on satisfaction in wine tourism. At present, an increasing number of people and families are seeking esthetic places for relaxation and family reunions, particularly amid COVID-19. Rural tourism possesses such functions; it remains a novel phenomenon for visitors who live in urban areas and provides leisure and educational benefits when visitors to a rural site contemplate the landscape or participate in an agricultural process for leisure purposes (WTO, 2020). Tourists can obtain leisure and educational benefits, including ecological knowledge, information about green consumerism, leisure and recreational opportunities, health and food security, reduced mental health issues, and nostalgia nurturing (Alford and Jones, 2020; Ambelu et al., 2018; Christou, 2020; Lane, 1994; Li et al., 2021). These four perspectives possess a potential synergy, and their effects could strengthen the relationship between rural families and rural areas and stimulate new regional resilience. Therefore, rural tourism should be understood as an enabler of rural community development that will eventually attract policy-makers and stakeholders to invest more money in developing or advancing it.

## Methodology

**Fig. 1:**
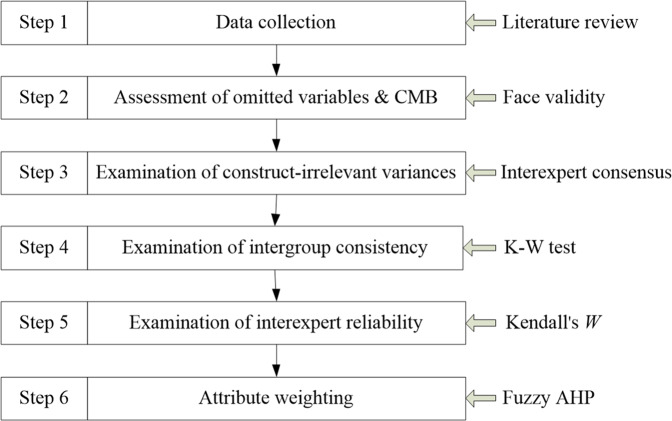
Model development procedure. Steps required in developing the model for analysis after obtaining the data.

The literature on rural tourism provides no generally accepted method for measuring its contributions or sustainability intensity. Although many statistical methods are available, several limitations remain, particularly in terms of the item generation stage and common method bias (CMB). For example, Marzo-Navar et al. (2015) used the mean and SD values to obtain their items. However, the use of the mean has been criticized because it is susceptible to extreme values or outliers. In addition, they did not examine omitted variables and CMB. Asmelash and Kumar (2019) used the Delphi method with a mean value for deleting items. Although they asked experts to suggest the inclusion of any missed variables, they did not discuss these results. Moreover, they did not assess CMB. Islam et al. (2021) used a sixteen-step process to formulate sustainability indicators but did not consider omitted variables, a source of endogeneity bias. They also did not designate a priority for each indicator. Although a methodologically sound systematic review is commonly used, little attention has been given to reporting interexpert reliability when multiple experts are used to making decisions at various points in the screening and data extraction stages (Belur et al., 2021). Due to the limitations of the current methods for assessing sustainable tourism development, we aim to provide new methodological insights. Specifically, we suggest a six-stage procedure, as shown in Fig. 1.

Many sources of data collection can be used, including literature reviews, inferences about the theoretical definition of the construct, previous theoretical and empirical research on the focal construct, advice from experts in the field, interviews, and focus groups. In this study, the first step was to retrieve data from a critical literature review. The second step was the assessment of omitted variables to produce items that fully captured all essential aspects of the focal construct domain. In this case, researchers must not omit a necessary measure or fail to include all of the critical dimensions of the construct. In addition, the stimuli of CMB, for example, double-barreled items, items containing ambiguous or unfamiliar terms, and items with a complicated syntax, should be simplified and made specific and concise. That is, researchers should delete items contaminated by CMB. The third step was the examination of construct-irrelevant variance to retain the variances relevant to the construct of interest and minimize the extent to which the items tapped concepts outside the focal construct domain. Variances irrelevant to the targeted construct should be deleted. The fourth step was to examine intergroup consistency to ensure that there was no outlier impact underlying the ratings. The fifth step was to examine interexpert reliability to ensure rating conformity. Finally, we prioritized the importance of each variable with the fuzzy analytic hierarchy process (AHP), which is a multicriteria decision-making approach. All methods used in this study are expert-based approaches.

### Selection of experts

Because this study explores the contributions of rural tourism to rural community development, it involves phenomena in the postdevelopment stage; therefore, a few characteristics are essential for determining the choice of experts. The elements used to identify the experts in this study were (1) the number of experts, (2) expertise, (3) knowledge, (4) diversity, (5) years working in this field, and 5) commitment to participation. Regarding the number of experts, Murphy-Black et al. (1998) suggested that the more participants there are, the better, as a higher number reduces the effects of expert attrition and rater bias. Taylor-Powell (2002) pointed out that the number of participants in an expert-based study depends not only on the purpose of the research but also on the diversity of the target population. Okoli and Pawlowski (2004) recommended a target number of 10–18 experts for such a purpose. Therefore, we recruited a group of 18 experts based on their stated interest in the topic and asked them to comment on our rationale concerning the rating priorities among the items. We asked them to express a degree of agreement or disagreement with each item we provided. We adopted a heterogeneous and anonymous arrangement to ensure that rater bias did not affect this study. The 18 experts had different backgrounds, which might have made it easier for them to reach a consensus objectively. We divided the eighteen experts into three subgroups: (1) at least six top managers from rural tourism businesses, all of whom had been in the rural tourism business for over 10 years; (2) at least six academics who taught subjects related to tourism at three different universities in Taiwan; and (3) at least six government officials involved in rural development issues in Taiwan.

### Generating items to represent the construct

#### Step 1: Data collection

Data collection provides evidence for investigation and reflects the construct of interest. While there is a need to know what rural tourism contributes, previous studies have provided no evidence for policy-makers to establish a rural community strategy; thus, it is essential to use a second source to achieve this aim. We used a literature review for specific topics; the data we used were based on the findings being presented in papers on rural tourism indexed in the SSCI (Social Sciences Citation Index) and SCIE (Science Citation Index Expanded). In this study, we intended to explore the role of rural tourism and its contributions to rural development. Therefore, we explored the secondary literature on the state of the questions of rural development, sustainable development, sustainability indicators, regional resilience, farm tourism, rural tourism, COVID-19, tourist preferences, and ecotourism using terms such as land ethics, ecology, biodiversity, green consumerism, environmentalism, green chemistry, community identity, community integration, community visibility, and development goals in an ad hoc review of previous studies via Google Scholar. Based on the outcomes of this first data collection step, we generated thirty-three subattributes and classified them into four domains.

#### Step 2: Examine the face validity of omitted variables and CMB

Face validity is defined as assessing whether a measurement scale or questionnaire includes all the necessary items (Dempsey and Dempsey, 1992). Based on the first step, we generated data subattributes from our literature review. However, there might have been other valuable attributes or subattributes that were not considered or excluded. Therefore, our purposes for examining face validity were twofold. First, we assessed the omitted variables, defined as the occurrence of crucial aspects or facets that were omitted (Messick, 1995). These comprise a threat to construct validity that, if ignored by researchers, might result in unreliable findings. In other words, face validity is used to distinguish whether the researchers have adequately captured the full dimensions of the construct of interest. If not, the evaluation instrument or model is deficient. However, the authors found that most rural tourism studies have not assessed the issue of omitted variables (An and Alarcon, 2020; Lin, 2022). Second, we mitigated the CMB effect. In a self-report survey, it is necessary to provide a questionnaire without CMB to the targeted respondents, as CMB affects respondent comprehension. Therefore, we assessed item characteristic effects, item context effects, and question response process effects. These three effects are related to the respondents’ understanding, retrieval, mood, affectivity, motivation, judgment, response selection, and response reporting (Podsakoff et al., 2003). Specifically, items containing flaws from these three groups in a questionnaire can seriously influence an empirical investigation and potentially result in misleading conclusions. We assessed face validity by asking all the experts to scrutinize the content items that we collected from the literature review and the questionnaire that we drafted. The experts could then add any attribute or subattribute they thought was essential that had been omitted. They could also revise the questionnaire if CMB were embedded. We added the new attributes or subattributes identified by the experts to those collected from the literature review.

#### Step 3: Examine interexpert consensus for construct-irrelevant variances

After examining face validity, we needed to rule out items irrelevant to the construct of interest; otherwise, the findings would be invalid. We examined the interexpert consensus to achieve this aim. The purpose was to estimate the experts’ ratings of each item. In other words, interexpert consensus assesses the extent to which experts make the same ratings (Kozlowski and Hattrup, 1992; Northcote et al., 2008). In prior studies, descriptive statistics have often been used to capture the variability among individual characteristics, responses, or contributions to the subject group (Landeta, 2006; Roberson et al., 2007). Many expert-based studies have applied descriptive statistics to determine consensus and quantify its degree (Paraskevas and Saunders, 2012; Stewart et al., 2016). Two main groups of descriptive statistics, central tendencies (mode, mean, and median) and level of dispersion (standard deviation, interquartile, and coefficient of variation), are commonly used when determining consensus (Mukherjee et al., 2015). Choosing the cutoff point of interexpert consensus was critical because we used it as a yardstick for item retention and its value can also be altered by a number on the Likert scale (Förster and von der Gracht, 2014). In the case of a 5-point Likert scale, the coefficient of variation (CV) is used to measure interexpert consensus. Hence, CV ≤ 0.3 indicated high consensus (Zinn et al., 2001). In addition, based on the feedback obtained from the expert panel, we used standard deviation (SD) as another measurement to assess the variation in our population. Henning and Jordaan (2016) indicate that SD ≤ 1 represents a high level of consensus, meaning that it can act as a guideline for cutoff points. In addition, following Vergani et al. (2022), we used the percentage agreement (% AGR) to examine interexpert consensus. If the responses reached ≧70% 4 and 5 in the case of a 5-point Likert scale, it indicated that the item had interexpert consensus; thus, we could retain it. Moreover, to avoid the impact of outliers, we used the median instead of the mean as another measurement. Items had a high consensus if their median value was ≥4.00 (Rice, 2009). Considering these points, we adopted % AGR, median, SD, and CV to examine interexpert consensus.

#### Step 4: Examine intergroup consistency

In this expert-based study, the sample size was small. Any rater bias could have caused inconsistency among the subgroups of experts; therefore, we needed to examine the effect of rater bias on intergroup consistency. When the intergroup ratings showed substantially different distributions, the aggregated data were groundless. Dajani et al. (1979) remarked that interexpert consensus is meaningless if the consistency of responses in a study is not reached, as it means that any rater bias could distort the median, SD, or CV. Most studies have used one-way ANOVA to determine whether there is a significant difference between the expected and observed frequency in three or more categories. However, this method is based on large sample size and normal distribution. In the case of expert-based studies, the expert sample size is small, and the assessment distribution tends to be skewed. Thus, we used the nonparametric test instead of one-way ANOVA for consistency measurement (Potvin and Roff, 1993). We used the Kruskal‒Wallis test (K–W) to test the intergroup consistency among the three subgroups of experts. The purpose of the K–W test is to determine whether there are significant differences among three or more subgroups regarding the ratings of the domains (Huck, 2004). The judgment criteria in the K-W test depended on the level of significance, and we set the significance level at *p* < 0.05 (Love and Irani, 2004), with no significant differences among groups set at *p* > 0.05 (Loftus et al., 2000; Rice, 2009). We used SPSS to conduct the K–W test to assess intergroup consistency in this study.

#### Step 5: Examine interexpert reliability

Interexpert reliability, on the one hand, is usually defined as the proportion of systematic variance to the total variance in ratings (James et al., 1984). On the other hand, interexpert reliability estimation is not concerned with the exact or absolute value of ratings. Rather, it measures the relative ordering or ranking of rated objects. Thus, interexpert reliability estimation concerns the consistency of ratings (Tinsley and Weiss, 1975). If an expert-based study did not achieve interexpert reliability, we could not trust its analysis (Singletary, 1994). Thus, we examined interexpert reliability in this expert-based study. Many methods are available in the literature for measuring interexpert reliability, but there seems to be little consensus on a standard method. We used Kendall’s *W* to assess the reliability among the experts for each sample group (Goetz et al., 1994) because it was available for any sample size or ordinal number. If *W* was 1, all the experts were unanimous, and each had assigned the same order to the list of objects or concerns. As Spector et al. (2002) and Schilling (2002) suggested, reliabilities well above the recommended value of .70 indicate sufficient internal reliability. In this study, there was a strong consensus when *W* > 0.7. *W* > 0.5 represented a moderate consensus; and *W* < 0.3 indicated weak interexpert agreement (Schmidt et al., 2001). To measure Kendall’s *W*, we used SPSS 23 to assess interexpert reliability.

#### Step 6: Examine the fuzzy analytic hierarchy process

After examining face validity, interexpert consensus, intergroup consistency, and interexpert reliability, we found that the aggregated items were relevant, authentic, and reliable in relation to the construct of interest. To provide policy-makers with a clear direction regarding which contributions are more or less important, we scored each attribute and subattribute using a multicriteria decision-making technique. Fuzzy AHP is a well-known decision-making tool for modeling unstructured problems. It enables decision-makers to model a complex issue in a hierarchical structure that indicates the relationships between the goal, criteria, and subcriteria on the basis of scores (Park and Yoon, 2011). The fuzzy AHP method tolerates vagueness and ambiguity (Mikhailov and Tsvetinov, 2004). In other words, fuzzy AHP can capture a human’s appraisal of ambiguity when considering complex, multicriteria decision-making problems (Erensal et al., 2006). In this study, we used Power Choice 2.5 software to run fuzzy AHP, determine weights, and develop the impact structure of rural tourism on sustainable rural development.

## Results

### Face validity

To determine whether we had omitted variables, we asked all 18 experts to scrutinize our list of four attributes and 33 subattributes for omitted variables and determine whether the questionnaire contained any underlying CMB. We explained the meaning of omitted variables, the stimuli of CMB, and the two purposes of examining face validity to all the experts. In their feedback, the eighteen experts added one item as an omitted variable: business viability. The experts suggested no revisions to the questionnaire we had drafted. These results indicated that one omitted variable was revealed and that our prepared questionnaire was clear, straightforward, and understandable. The initially pooled 34 subattributes represented the construct of interest, and all questionnaires used for measurement were defendable in terms of CMB. The biasing effects of method variance did not exist, indicating that the threat of CMB was minor.

### Interexpert consensus

In this step, we rejected any items irrelevant to the construct of interest. Consensus measurement played an essential role in aggregating the experts’ judgments. This study measured the AGR, median, SD, and CV. Two items, strategic alliance (AGR = 50%) and carbon neutrality (AGR = 56%) were rated < 70%, and we rejected them accordingly. These results are shown in Table 1. The AGR, median, SD, and CV values were all greater than the cutoff points, thus indicating that the majority of experts in this study consistently recognized high values and reached a consensus for the rest of the 32 subattributes. Consequently, the four attributes and 32 subattributes remained and were initially identified as determinants for further analysis.

**Table 1:** Results of interexpert consensus.

Indicator	% AGR	Median	SD	CV
A. *Economic perspective*				
A_1_. Investment opportunities	77	4.0	0.74	0.18
A_2_. Increased income	72	4.0	0.71	0.19
A_3_. Employment opportunities	83	4.0	0.69	0.17
A_4_. Employment stability	72	4.0	0.65	0.17
A_5_. Tax revenue	72	4.0	0.81	0.21
A_6_. Entrepreneurial opportunities	77	4.5	0.75	0.17
A_7_. Economic resilience	72	4.0	0.75	0.20
A_8_. Strategic alliance	*50*	3.5	0.77	0.23
A_9_. Product diversification	72	4.0	0.71	0.20
A_10_. Business viability	72	4.0	0.74	0.18
B. *Sociocultural perspective*				
B_1_. Quality of life & well-being	89	5.0	0.68	0.15
B_2_. Depopulation	89	4.5	0.68	0.16
B_3_. Social stability	72	4.0	0.83	0.23
B_4_. Culture & heritage preservation	72	4.0	0.71	0.18
B_5_. Restoration of historical buildings	77	4.0	0.63	0.16
B_6_. Community identity	83	4.5	0.75	0.17
B_7_. Community pride	72	4.0	0.91	0.24
B_8_. Community visibility	72	4.0	0.80	0.22
B_9_. Cultural integrity	72	4.0	0.75	0.10
C. *Environmental perspective*				
C_1_. Natural environmental conservation	77	4.0	0.60	0.14
C_2_. Biodiversity	72	4.0	0.80	0.22
C_3_. Infrastructure	83	5.0	0.76	0.10
C_4_. Green chemistry	72	4.0	0.53	0.14
C_5_. Carbon neutrality	*56*	4.0	0.76	0.22
C_6_. Kept land unspoiled	72	4.0	0.80	0.22
C_7_. Kept land in family	83	4.0	0.85	0.20
C_8_. Environmental awareness	72	4.0	0.65	0.17
D. *Leisure and educational perspective*				
D_1_. Ecological knowledge	83	4.0	0.66	0.16
D_2_. Green consumerism	77	4.0	0.66	0.16
D_3_. Technology skills and capabilities	77	4.0	0.49	0.12
D_4_. Leisure and recreational opportunities	83	4.5	0.75	0.17
D_5_. Health and food security	77	4.0	0.66	0.17
D_6_. Reducing mental health issues	77	4.0	0.65	0.15
D_7_. Nostalgia nurturing	72	4.0	0.75	0.18

Cutoff points on a 5-point Likert scale: AGR ≥ 70%, median ≥ 4, SD ≤ 1, and CV ≤ 0.3.

The italic values indicate that they are out of the cut-off point and should be rejected accordingly.

### Intergroup consistency and interexpert reliability

In this study, with scores based on a 5-point Likert scale, we conducted the K–W test to assess intergroup differences for each subattribute. Based on the outcomes, the K–W test yielded significant results for all 32 subattributes; all three groups of experts reached consistency at *p* > 0.05. This result indicated that no outlier or extreme value underlay the ratings, and therefore, intergroup consistency was reached. Finally, we measured interexpert reliability with Kendall’s *W*. The economic perspective was *W* = 0.73, the sociocultural perspective was *W* = 0.71, the environmental perspective was *W* = 0.71, and the leisure and educational perspective was *W* = 0.72. These four groups of *W* were all ≧0.7, indicating high reliability for the ranking order and convergence judged by all subgroup experts. These results are shown in Table 2.

**Table 2:** Results of intergroup consistency and interexpert reliability analysis.

Indicator	K–W *X*^2^	*p*	Kendall’s *W*
A. *Economic perspective*			0.73
A_1_. Investment opportunities	0.68	0.336	
A_2_. Increased income	1.40	0.258	
A_3_. Employment opportunities	0.87	0.308	
A_4_. Employment stability	0.67	0.343	
A_5_. Tax revenue	2.48	0.146	
A_6_. Entrepreneurial opportunities	1.68	0.216	
A_7_. Economic resilience	1.22	0.234	
A_8_. Product diversification	2.05	0.128	
A_9_. Business viability	2.76	0.092	
B. *Sociocultural perspective*			0.71
B_1_. Quality of life & well-being	0.87	0.312	
B_2_. Depopulation	0.78	0.328	
B_3_. Social stability	0.53	0.364	
B_4_. Culture & heritage preservation	0.84	0.325	
B_5_. Restoration of historical buildings	1.57	0.128	
B_6_. Community identity	0.82	0.312	
B_7_. Community pride	2.06	0.118	
B_8_. Community visibility	1.76	0.106	
B_9_. Cultural integrity	2.56	0.095	
C. *Environmental perspective*			0.71
C_1_. Natural environmental conservation	1.58	0.131	
C_2_. Biodiversity	0.82	0.292	
C_3_. Infrastructure	1.76	0.106	
C_4_. Green chemistry	0.78	0.328	
C_5_. Kept land unspoiled	1.22	0.234	
C_6_. Kept land in family	0.64	0.355	
C_7_. Environmental awareness	2.35	0.094	
D. *Leisure and educational perspective*			0.72
D_1_. Ecological knowledge	1.78	0.098	
D_2_. Green consumerism	1.13	0.218	
D_3_. Technology skills and capabilities	0.68	0.336	
D_4_. Leisure & recreational opportunities	0.64	0.356	
D_5_. Health & food security	1.04	0.234	
D_6_. Reducing mental health issues	0.75	0.340	
D_7_. Nostalgia nurturing	1.42	0.154	

*p* > 0.05.

### The hierarchical framework

**Fig. 2:**
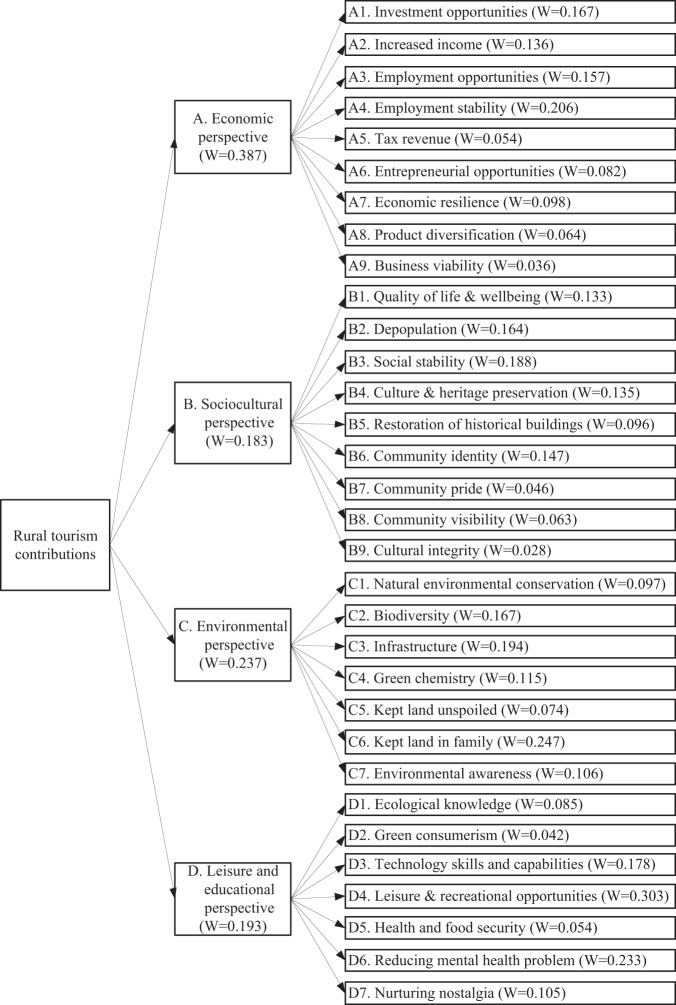
Rural tourism contribution model. The priority index of each attribute and sub-attribute.

The results of this study indicate that rural tourism contributions to rural community development comprise four attributes and thirty-two subattributes. The economic perspective encompasses nine subattributes and is weighted at *w* = 0.387. In addition, rural tourism has long been considered a possible means of sociocultural development and regeneration of rural areas, particularly those affected by the decline in traditional rural

activities, agricultural festivals, and historical buildings. According to the desired benefits, the sociocultural perspective encompasses nine subattributes and is weighted at *w* = 0.183. Moreover, as rural tourism can develop on farms and locally, its contribution to maintaining and enhancing environmental regeneration and protection is significant. Therefore, an environmental perspective can determine rural tourism’s impact on pursuing environmental objectives. Our results indicate that the environmental perspective encompasses seven subattributes and that its weight is *w* = 0.237. Furthermore, the leisure and educational perspective indicates the attractiveness of rural tourism from visitors’ viewpoint and their perception of a destination’s value and contributions. These results show that this perspective encompasses seven subattributes and is weighted at *w* = 0.193. This specific contribution model demonstrates a 3-level hierarchical structure, as shown in Fig. 2. The scores for each criterion could indicate each attribute’s importance and explain the priority order of the groups. Briefly, the critical sequence of each measure in the model at Level 2 is as follows: economic perspective > environmental perspective > leisure and educational perspective > sociocultural perspective. Since scoring and ranking were provided by 18 experts from three different backgrounds and calculated using fuzzy AHP, our rural tourism contribution model is established. It can provide policy-makers with information on the long-term benefits and advantages following the completion of excellent community development in rural areas.

## Discussion/Implications

In the era of sustainable rural development, it is vital to consider the role of rural tourism and how research in this area shapes access to knowledge on rural community development. This study provides four findings based on the increasing tendency of policy-makers to use such information to shape their policy-making priorities. It first shows that the demand for rural tourism has soared, particularly during COVID-19. Second, it lists four significant perspectives regarding the specific contributions of rural tourism to rural community development and delineates how these four perspectives affect rural tourism development. Our findings are consistent with those of prior studies. For example, geography has been particularly important in the rural or peripheral tourism literature (Carson, 2018). In terms of the local geographical context, two contributions could be made by rural tourism. The first stems from the environmental perspective. When a rural community develops rural tourism, environmental protection awareness is increased, and the responsible utilization of natural resources is promoted. This finding aligns with Lee and Jan (2019). The second stems from the leisure and educational perspective. The geographical context of a rural community, which provides tourists with geographical uniqueness, advances naturally calming, sensory-rich, and emotion-generating experiences for tourists. These results suggest that rural tourism will likely positively impact tourists’ experience. This finding is consistent with Kastenhoz et al. (2020). Third, although expert-based approaches have considerable benefits in developing and testing underlying phenomena, evidence derived from interexpert consensus, intergroup consistency, and interexpert reliability has been sparse. This study provides such evidence. Fourth, this research shows that rural tourism makes four main contributions, economic, sociocultural, environmental, leisure, and educational, to rural community development. Our results show four key indicators at Level 2. The economic perspective is strongly regarded as the most important indicator, followed by the environmental perspective, leisure and educational perspective, and sociocultural perspective, which is weighted as the least important. The secondary determinants of contributions have 32 subindicators at Level 3: each was identified and assigned a different weight. These results imply that the attributes or subattributes with high weights have more essential roles in understanding the contributions of rural tourism to rural community development. Policy-makers can use these 32 subindicators to formulate rural tourism development policies or strategies.

This study offers the following five practical implications for policymakers and rural communities:

First, we argue that developing rural tourism within a rural community is an excellent strategy for revitalization and countering the effects of urbanization, depopulation, deforestation, and unemployment.

Second, our analytical results indicate that rural tourism’s postdevelopment contribution is significant from the economic, sociocultural, environmental, leisure, and educational perspectives, which is consistent with Lee and Jan (2019).

Third, there is an excellent opportunity to build or invest more in rural tourism during COVID-19, not only because of the functions of rural tourism but also because of its timing. Many prior studies have echoed this recommendation. For example, Yang et al. (2021) defined rural tourism as the leading industry in rural areas, offering an output value ten times higher than that of agriculture in China. In addition, rural tourism has become more attractive to urban tourists amid COVID-19. Vaishar and Šťastná (2022) suggested that the COVID-19 pandemic created a strong demand for rural tourism, which can mitigate threats to public mental health, such as anxiety, depression, loneliness, isolation, and insomnia. Marques et al. (2022) showed that tourists’ preference for tourism in rural areas increased substantially during COVID-19.

Fourth, the contributions of this study to policy development are substantial. The more focused rural tourism in rural areas is, the more effective revitalization becomes. This finding highlights the importance of such features in developing rural tourism to enhance rural community development from multiple perspectives. This finding echoes Zawadka et al. (2022); i.e., policy-makers should develop rural tourism to provide tourists with a safe and relaxed environment and should not ignore the value of this model for rural tourism.

Fifth, our developed model could drive emerging policy issues from a supporting perspective and provide policy-makers with a more comprehensive overview of the development of the rural tourism sector, thus enabling them to create better policies and programs as needed. For example, amid COVID-19, rural tourism created a safe environment for tourists, mainly by reducing their fears of contamination (Dennis et al., 2021). This novel contribution that rural tourism destinations can provide to residents and visitors from other places should be considered and built into any rural community development policy.

This study also has the following four methodological implications for researchers:

First, it addresses methodological limitations that still impede tourism sustainability model development. Specifically, we suggest a six-stage procedure as the guideline; it is imperative that rural tourism researchers or model developers follow this procedure. If they do not, their findings tend to be flawed.

Second, to ensure that collected data are without extraneous interference or differences via subgroups of experts, the assessment of intergroup consistency with the K–W test instead of one-way ANOVA is proposed, especially in small samples and distribution-free studies.

Third, providing interexpert reliability evidence within expert-based research is critical; we used Kendall’s *W* to assess the reliability among experts for each sample group because it applies to any sample size and ordinal number.

Finally, we recommend using fuzzy AHP to establish a model with appropriate indicators for decision-making or selection. This study offers novel methodological insights by estimating a theoretically grounded and empirically validated rural tourism contribution model.

There are two limitations to this study. First, we examine all subattributes by interexpert consensus to delete construct-irrelevant variances that might receive criticism for their lack of statistical rigor. Future studies can use other rigorous methods, such as AD_M(*j*)_ or rWG_(*j*)_, interexpert agreement indices to assess and eliminate construct-irrelevant variances. Second, we recommend maximizing rural tourism contributions to rural community development by using the general population as a sample to identify any differences. More specifically, we recommend using Cronbach’s alpha, confirmatory factor analysis (CFA), and structural equation modeling (SEM) to test the overall reliability and validity of the data and results. It is also necessary to provide results for goodness-of-fit measures—e.g., the goodness-of-fit index (GFI), adjusted goodness-of-fit index (AGFI), comparative fit index (CFI), normed fit index (NFI), Tucker–Lewis Index (TLI), standardized root mean square residual (SRMR), or root mean square error of approximation (RMSEA).

## Conclusion

Numerous empirical studies have illustrated how rural tourism can positively and negatively affect the contexts in rural areas where it is present. This study reveals the positive contributions of rural tourism to rural community development. The findings show that using rural tourism as a revitalization strategy is beneficial to nonurban communities in terms of their economic, sociocultural, environmental, and leisure and educational development. The contribution from the economic perspective is particularly important. These findings suggest that national, regional, and local governments or community developers should make tourism a strategic pillar in their policies for rural development and implement tourism-related development projects to gain 32 benefits, as indicated in Fig. 2. More importantly, rural tourism was advocated and proved effective for tourists and residents to reduce anxiety, depression, or insomnia during the COVID-19 pandemic. With this emerging contribution, rural tourism is becoming more critical to tourists from urban areas and residents involved in rural community development. With this model, policy-makers should not hesitate to develop or invest more in rural communities to create additional tourism-based activities and facilities. As they could simultaneously advance rural community development and public mental health, policy-makers should include these activities among their regional resilience considerations and treat them as enablers of sustainable rural development. We conclude that amid COVID-19, developing rural tourism is an excellent strategy for promoting rural community development and an excellent alternative that could counteract the negative impacts of urbanization and provide stakeholders with more positive interests. The proposed rural tourism contribution model also suggests an unfolding research plan.

## Data Availability

The datasets generated and/or analyzed during the current study are available from the corresponding author on reasonable request.
